# A 3D printing approach toward targeted intervention in telerehabilitation

**DOI:** 10.1038/s41598-020-59927-y

**Published:** 2020-02-28

**Authors:** Roni Barak Ventura, Alessandro Rizzo, Oded Nov, Maurizio Porfiri

**Affiliations:** 1 Department of Mechanical and Aerospace Engineering, New York University Tandon School of Engineering, Brooklyn, New York 11201 USA; 2 Dipartimento di Elettronica e Telecomunicazioni, Politecnico di Torino, Corso Duca degli Abruzzi 24, Torino, 10129 Italy; 3 Office of Innovation, New York University Tandon School of Engineering, Brooklyn, New York 11201 USA; 4 Department of Technology Management and Innovation, New York University Tandon School of Engineering, 5 MetroTech Center, Brooklyn, New York 11201 USA; 5 Department of Biomedical Engineering, New York University Tandon School of Engineering, Brooklyn, New York 11201 USA

**Keywords:** Health care, Preclinical research

## Abstract

Neuromuscular impairment requires adherence to a rehabilitation regimen for maximum recovery of motor function. Consumer-grade game controllers have emerged as a viable means to relay supervised physical therapy to patients’ homes, thereby increasing their accessibility to healthcare. These controllers allow patients to perform exercise frequently and improve their rehabilitation outcomes. However, the non-universal design of game controllers targets healthy people and does not always accommodate people with disability. Consequently, many patients experience considerable difficulty assuming certain hand postures and performing the prescribed exercise correctly. Here, we explore the feasibility of improving rehabilitation outcomes through a 3D printing approach that enhances off-the-shelf game controllers in home therapy. Specifically, a custom attachment was 3D printed for a commercial haptic device that mediates fine motor rehabilitation. In an experimental study, 25 healthy subjects performed a navigation task, with the retrofit attachment and without it, while simulating disability of the upper limb. When using the attachment, subjects extended their wrist range of motion, yet maintained their level of compensation. The subjects also showed higher motivation to repeat the exercise with the enhanced device. The results bring forward evidence for the potential of this approach in transforming game controllers toward targeted interventions in home therapy.

## Introduction

Neurological disorders often lead to profound disability that is associated with a loss of mobility and independence, adversely impacting all facets of life^1,2^. It is estimated that more than 11 million Americans require assistance to perform activities of daily living, costing at least $170 billion every year^3^. Approximately half of these costs are indirect, stemming from unemployment and loss of productivity^3^. Individuals with neuromuscular disability can be successfully reintegrated into society by adhering to a rehabilitation regimen consisting of repetitive, high-intensity exercises^4–6^. Nonetheless, the vast majority of patients do not receive treatment with sufficient frequency to achieve this goal. The process of leaving their homes and traveling is often exceedingly incommodious, impeding them from arriving at a clinic^7–9^. Moreover, the rising costs of treatments, understaffing of medical specialists, and long waiting periods prevent them access to out-patient care. Therefore, patients’ recovery usually depends on the performance of exercise at home, with limited professional feedback^5^.

Telerehabilitation offers an effective means for the administration of affordable and convenient physical therapy to patients’ homes. In telerehabilitation, actuation and sensing technologies are utilized to relay physical therapy while providing medical specialists with objective performance scores to remotely gauge the progress of recovery. Telerehabilitation is advantageous for several reasons. First, it eliminates the imposition associated with traveling^7,9^. Second, being able to perform therapeutic exercise comfortably at home, patients can accommodate their personal schedule such that long waiting times for treatment are prevented^7^. Third, telerehabilitation allows therapists to provide services for multiple patients simultaneously, and supplement the exercise with frequent feedback^10,11^. While all of these reasons favor telerehabilitation over traditional in-clinic therapy, telerehabilitation remains impracticable due to high costs^10,12^.

Reducing the costs of telerehabilitation is possible by adopting commercial devices^12,13^. In this vein, consumer-grade gaming devices have been increasingly repurposed to administer physical therapy. Java Therapy is an early example of a web-based, low-cost telerehabilitation platform, targeting upper limb impairment in stroke patients^14^. It consists of a haptic Logitech Wingman Forcefeedback Pro joystick and a custom computer software. Although tested only on a single patient, this platform was demonstrated to reliably measure various metrics, such as coordination and strength, and improve coordination and motor control, whereby the patient moved the joystick more smoothly and more precisely by the end of a 12-week period. Remote hand-held controllers have also been used in telerehabilitation, allowing for exercise of both gross and fine motor skills. For example, the Sony PlayStation II EyeToy has been shown to improve postural control and functional mobility, and the Nintendo Wii led to improved grip strength and overall motor function^15,16^. Therefore, it seems tenable that impairments can be treated with commercial game controllers of sorts.

In spite of their promising telerehabilitative prospects, off-the-shelf devices are not free of limitations. Principally, these limitations can be divided into two categories, one psychological and another physical. The former involves user experience, where telerehabilitation interfaces present tasks in a repetitive manner and compromises patients’ ability to sustain motivation for a prolonged period of time^17^. The latter concerns the limited ability of the device to promote healthy physiological movements while inhibiting the execution of abnormal movements^18^.

To address low adherence due to interaction design, a large number of studies investigated motivational interventions to improve engagement in telerehabilitation. Among them, many employed gamification strategies, such as story-telling, displaying performance scores, or incorporating competitive elements^19–21^. For example, the developers of Java Therapy acknowledged that physical therapy is not intrinsically engaging, and created two components in the software. Between “Status Tests” that quantify movement, the patient had the option of voluntarily playing the classical “Breakout” game, which provided a recreational context to the therapy. These games could be further enriched through the incorporation of serious components and citizen science elements^10,22^. Such interventions may also cater to older patients, who constitute the majority of patients with neurological disability^19,23,24^.

Addressing physical limitations of commercial controllers is more problematic. Non-haptic devices offer a wide range of motions but are not able to direct motion into desired physiological movements^25^. Inadequate control over the patients’ movement could elicit compensatory movements^17,26^. Compensatory movements add degrees of freedom to patients’ kinematics by recruiting muscles that should not be involved in a movement^27^. For example, stroke patients tend to displace their trunk and scapula during reaching tasks to compensate for limited range of motion of their shoulder^18,27^. Such compensatory strategies pose a risk of injury, reinforce the use of non-physiological movements, and impede functional recovery. At the expense of a narrower workspace, haptic devices could provide force feedback and mitigate compensatory activities, although their success varies with the rehabilitation regimen and the pathology of the patient^12,28^.

Physical limitations of game controllers are inherent to their mass production, which reduces cost and broadens access to products. Mass production necessitates a global design and often does not support personalized design at a low cost. As a result, the rehabilitative features of commercial devices cannot be adapted for individuals’ pathologies and morphologies, and the benefits of a patient-centered treatment are curtailed. Modifying these controllers at a low price is warranted, especially for haptic devices that can implement different assistance strategies through force feedback^28^.

Additive manufacturing opens new avenues to overcome the invariability of global designs. It is commonly employed in medicine to personalize treatments, improve the outcomes of interventions, and reduce the costs of medical devices^13,29^. Additive manufacturing has already transformed several fields within rehabilitation medicine^30^. In orthopedics, prosthetic mandibles, skulls, noses, and ears are routinely 3D-printed^30–33^. Similarly, teeth, bones, and skin are layer-printed to produce tissue constructs^34–36^. However, in the physical therapy domain, additive manufacturing has been meagerly utilized, where in few instances exoskeletons for limb robotic rehabilitation were produced^37,38^. 3D printing could offer an additional mechanism toward patient-tailored telerehabilitation through personalized hardware.

**Figure 1:**
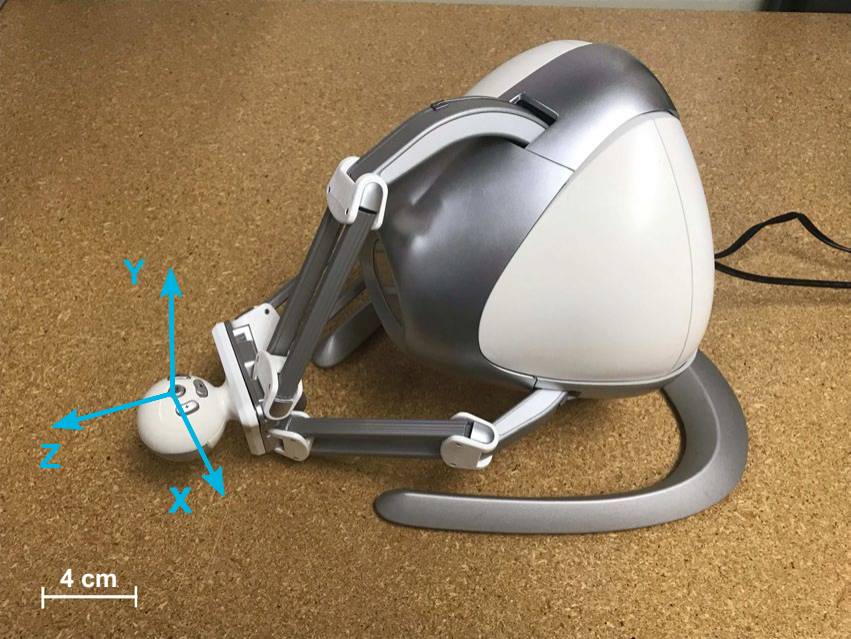
The Novint Falcon game controller with overlaid global coordinate system, describing the end-effector’s motion in three dimensions.

Herein, we propose to harness additive manufacturing technologies to supplement game controllers with custom-made attachments. We demonstrate the feasibility of this approach by retrofitting a controller for fine motor rehabilitation. We focus on the Novint Falcon (Novint Technologies, Albuquerque, New Mexico), a low-cost haptic device capable of delivering effective fine motor rehabilitation for the wrist and fingers^39–41^. The Novint Falcon (Fig. 1) provides translational motion along three axes with programmable sensory motor feedback^39^. While the Novint Falcon features are favorable for telerehabilitation, individuals with moderate to severe disability are not able to grasp the end-effector and manipulate it unassisted. Therefore, we customized a retrofit attachment that aimed at promoting wrist movement, while dampening compensatory movements.

We evaluated the usability of the attachment with healthy subjects, at a pre-clinical level that would not harm patients. The subjects simulated the physical disability resulting from a stroke using standard disability awareness training techniques^42^. Subjects manipulated the Novint Falcon to navigate through a maze^43^, and performed the navigation task twice, once with an augmented end-effector (experimental condition) and once with an unmodified end-effector (control condition).

We formulated three hypotheses.

(i) Subjects would improve their fine motor performance when using the attachment, reflected through a wider range of motion of the wrist and a higher variability of its angle – range of motion is a common metric of motor performance, measuring the isolation of movement^27,44^; angle variability conveys the extent to which the wrist would span its range of motion^45^.
(ii) The use of the attachment would reduce compensatory movement, reflected through a narrower range of motion of the trunk and lower variability of its displacement – these quantities are measured using a motion capture system.
(iii) The increased versatility offered by the retrofit attachment would increase users’ enjoyment and willingness to repeat the exercise.

Herein, we report the results, confirming the possibility of enhancing patients’ joint movement during rehabilitation exercise, using low-cost personalized end-effectors.

## Methods

### 3D-printed retrofit attachment

A retrofit attachment was designed for the Novint Falcon controller (Fig. 1). The Novint Falcon’s nominal dimensions are $228.3\,{\rm{mm}}\,\times \,228.3\,{\rm{mm}}\,\times \,228.3\,{\rm{mm}}$. Its end-effector can be moved within a $101.6\,{\rm{mm}}\,\times \,101.6\,{\rm{mm}}\,\times \,101.6\,{\rm{mm}}$ workspace with a maximum positional resolution of 400 dpi^39^. It can provide a force feedback of up to 8.8 N with a time response of 1 kHz, allowing for dampening of the end-effector toward assistive strategies with varying levels of difficulty^39,46^.

**Figure 2:**
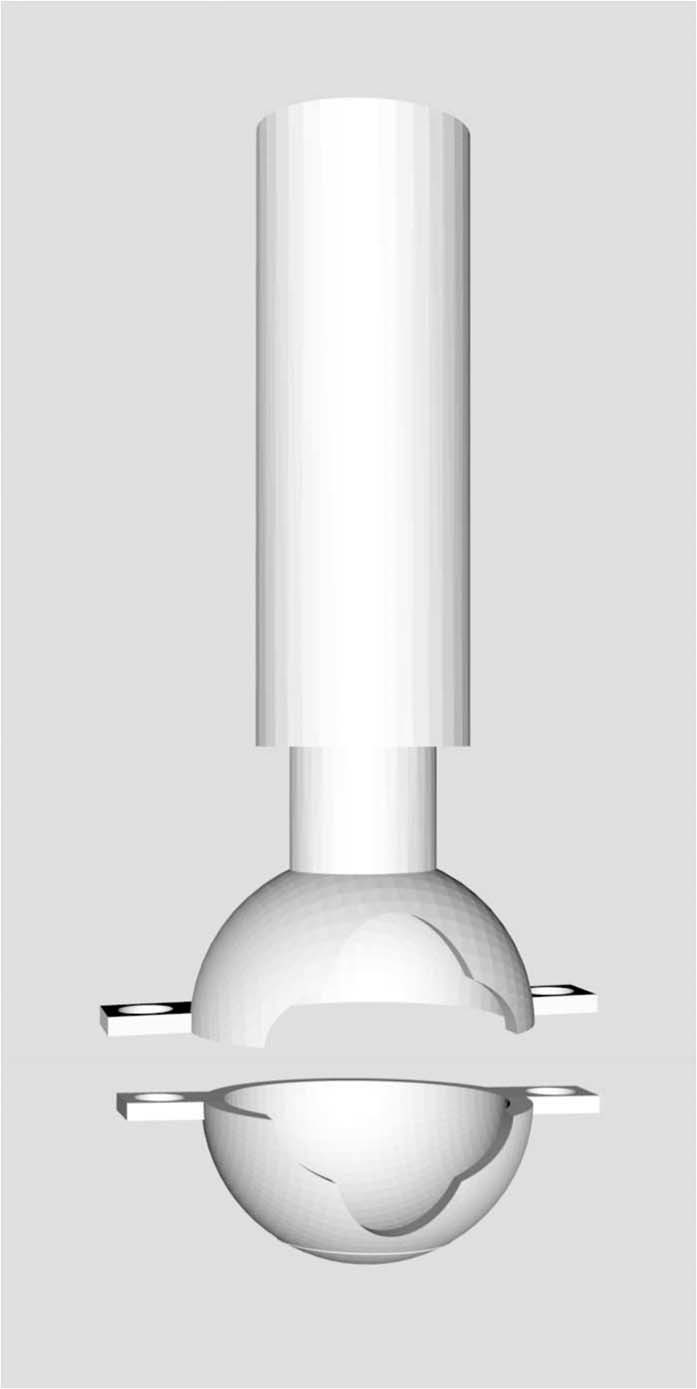
A computer-aided design of the retrofit attachment, dedicated to the Novint Falcon. The two parts are joined together to encapsulate the spherical end-effector. The circular cutouts necessitate rotation of the attachment around the end-effector, prompting movement of the wrist.

**Figure 3:**
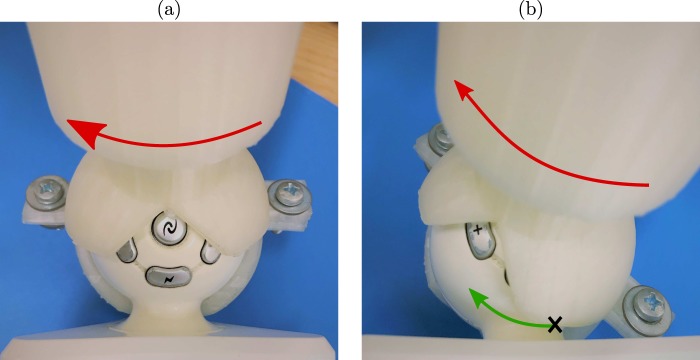
Movement of the end-effector with the retrofit attachment necessitates rotation of the wrist. The attachment begins in the neutral position, before the wrist has been rotated. (**a**) Rotational movement of the attachment, marked with the red arrow, does not influence the end-effector until its ridge reaches a point of contact with the end effector, marked with an X. (**b**) Rotational movement of the attachment now pushes the end effector (direction of motion marked with the green arrow).

Informed by pilot tests, a custom-made retrofit attachment was designed and refined in SolidWorks (Dassault Systèmes, Velizy-Villacoublay, France) to ease the grasp of the end effector and enhance wrist movement in fine motor rehabilitation^47^. The attachment consisted of a hollow sphere that can be lodged around the Novint Falcon’s end-effector with two bolts (Fig. 2). A vertical grab-bar extended from the sphere to enable a joystick-like grasp. The sphere’s radius was marginally larger than the end effector’s radius, creating a narrow space between the retrofit attachment and the end-effector interface such that their surfaces slid against one another. Four round cutouts were made on one side of the sphere to create an outlet where the delta robot’s legs connect to the end-effector. These cutouts were designed to promote wrist movement such that the user had to rotate their wrist further until the attachment was in contact with the neck of the end-effector and moved it (Fig. 3). Through this design, the attachment would effectively serve as a lever whereby a user would rotate their wrist to manipulate the end effector with reduced translational movement of the hand. Conceivably, the size of the slits could be adapted to create different levels of difficulty of manipulation. The final design was printed with the Ultimaker 2+ 3D printer (Ultimaker, Geldermalsen, The Netherlands).

### Experimental setup

**Figure 4:**
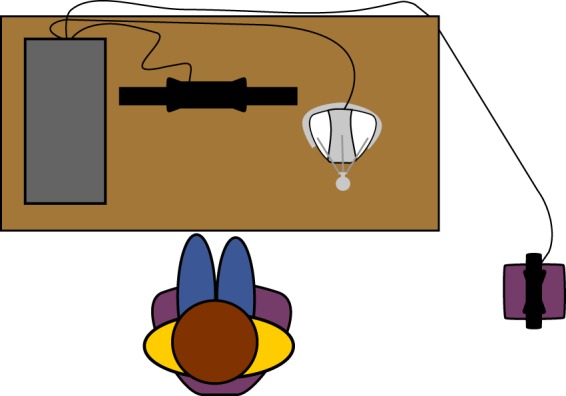
An illustration of the experimental setup from a top view. The user is seating in front of a desktop computer. The Novint Falcon is placed right of the computer screen. The Kinect is placed two meters on the right of the Novint Falcon, facing the user.

**Figure 5:**
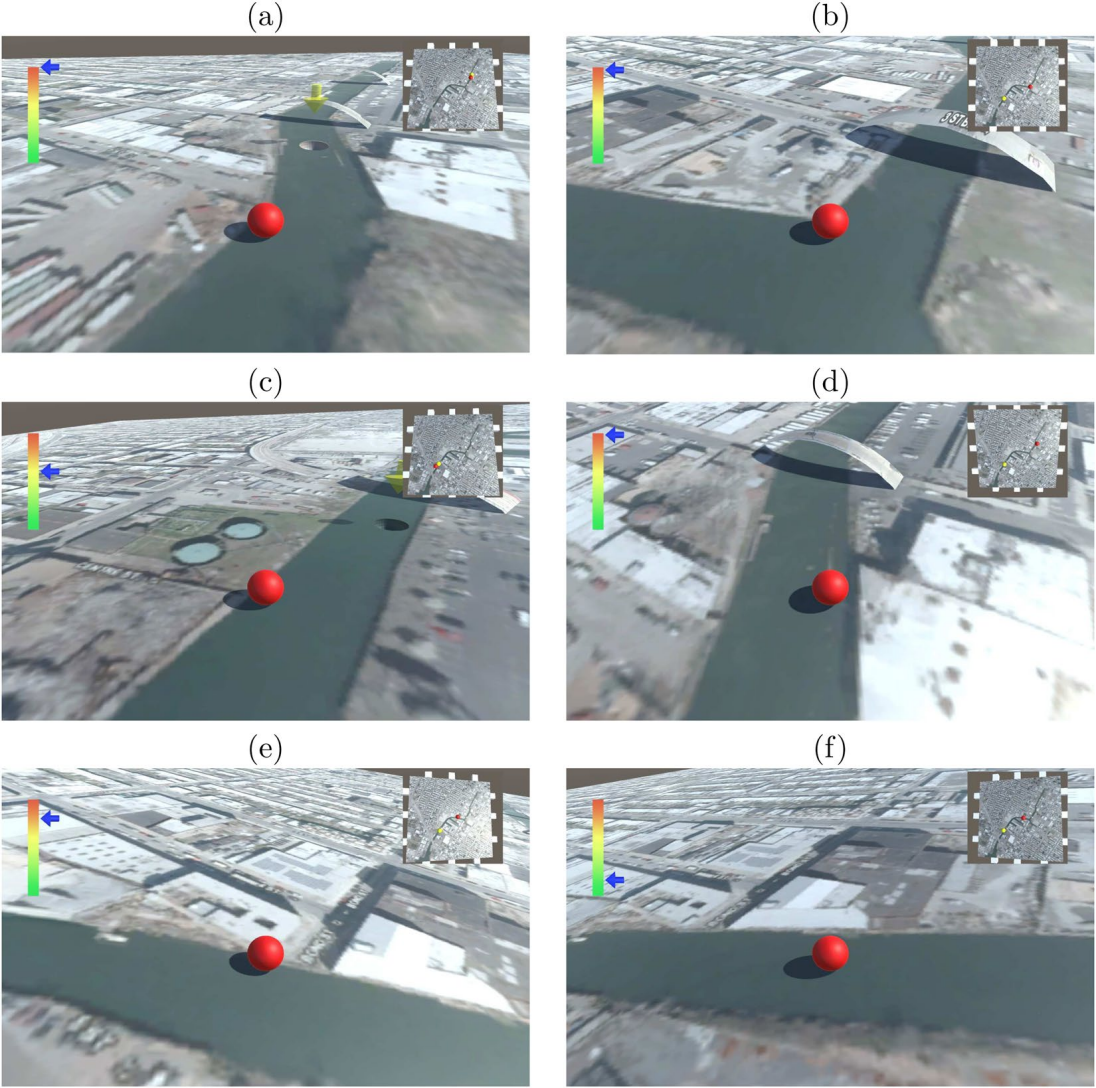
Snapshots of the navigation software. The objective of the navigation task is to maneuver a ball along a canal on a virtual plane toward a crater. (**a**) A miniature map on the upper-right screen delineated the location of the ball and the location of the crater. The ball starts from a balanced position. (**b**) To move the ball, the plane can be tilted toward north (**c**), south (**d**), east (**e**), and west (**f**).

The experimental setup consisted of a desktop computer, a Novint Falcon controller, and a Microsoft Kinect^TM^ sensor (Microsoft corporation, Redmond, Washington; Fig. 4). The Novint Falcon was interfaced with the desktop computer, to serve as a controller for a citizen science-based application^43^. In the application, users completed a navigation task where they maneuvered a red ball along a canal toward a basin on a virtual map, by tilting its plane (Fig. 5)^43^. Users tilted the virtual map toward north and south by moving the end-effector up and down along the $y$-axis, respectively. Similarly, users tilted the map toward west and east by moving the end-effector left and right along the $x$-axis, respectively.

The Kinect sensor was used to record compensatory movements reflected by trunk displacement. A computer vision color detection program was developed in the Kinect for Windows SDK, to track a bright blue tape, which would be placed along the participant’s trunk, extending from their left scapula to their waist. The sensor was placed two meters on the right of the Novint Falcon, facing toward the user from a lateral view.

### Experimental procedure

This study was approved by the institutional review board at New York University (IRB#FY2019-2828). Overall, 25 healthy volunteers were recruited on the New York University campus. Each volunteer was briefed by the experimenter on the experimental set-up, simulation of disability, and experimental procedure. Upon granting informed consent (where the volunteer also confirmed that they did not have any disability or a medical condition that might be exacerbated by the intervention), the preparation for the simulation of physical disability began^42,48,49^.

Each participant was fitted to a cold protection glove (Memphis Flex-Therm, MCR Safety, Collierville, Tennessee). A measuring tape was used to measure the dimensions of the participant’s hand, and their glove size was determined based on a standard glove size chart^50^. The participant wore the glove on their right, dexterous hand, and a wrist brace (ProFlex 675, Ergodyne Corporation, Saint Paul, Minnesota). The participant’s arm and trunk were wrapped together with bandages to limit shoulder movement^42^. Two inertial measurement units (IMU; MPU-6050, InvenSense Inc., Sunnyvale, California) were fixed in a 3D-printed housing and strapped onto the participant’s limb with an elastic drawstring. One IMU was strapped to the dorsum of their hand and the other was strapped to the dorsal side of their forearm.

**Figure 6:**
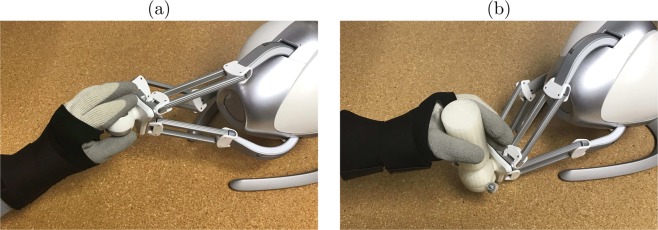
Experimental conditions with the Novint Falcon. Subjects manipulated the end-effector while wearing a thick glove and a wrist brace. They performed the experimental tasks twice, once with the unmodified end-effector (**a**), and once with the enhanced end-effector (**b**).

Once the gear was set up, the participant was seated in an armless chair in front of the computer. To calibrate the IMU sensors, the participant was asked to hold his/her forearm with the palm facing down, as horizontal to the ground as possible. To attain calibration, the participant performed supination followed by pronation of the forearm three times. Then, he/she proceeded to performing the navigation task twice, once with the retrofit attachment (experimental condition) and once without it (control condition) (Fig. 6). The order of the conditions was counterbalanced across users.

Upon completion of the navigation tasks, the participant removed the simulation apparatus. The participant completed a survey about their experience and preferences. Specifically, participants were asked to rate the extent to which they agree with four statements, using a 7-point Likert scale. The first statement was “I prefer using the Novint Falcon with the retrofit handle”. The second statement was “I enjoyed the activity better with the retrofit handle”. The third and fourth statements were “I would repeat this exercise daily with the retrofit handle” and “I would repeat this exercise daily without the retrofit handle”. After the responses were submitted, the experiment was concluded.

### Data analysis

Kinematic data were collected from two IMUs (referred as to IMU A and IMU B in the following) and the Kinect and processed in MatLab (MATLAB and Statistics Toolbox Release 2017^®^, The MathWorks, Inc., Natick, Massachusetts, United States). The processed data and survey responses were analyzed in R (R Core Team, Vienna, Austria^51^).

#### IMU data processing

**Figure 7:**
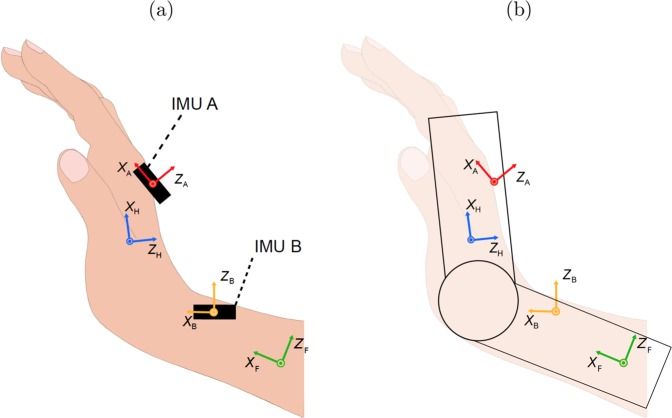
An illustration of IMU placement (represented by black rectangles) and the reference frames used in this study. One IMU was placed on the hand, and the other was placed on the forearm. (**a**) The reference frame for IMU $A$, IMU $B$, the hand, and the forearm are shown in red, orange, blue, and green, respectively. Limb coordinate systems were defined such that the $x$-axis pointed distally and $y$-axis was directed medially. The IMUs and the respective limb segments they are fastened to were regarded as a two rigid bodies, represented by two links connected through a revolute joint. (**b**) Misaligned IMUs would report false orientations for the limb segments. Therefore, a calibration procedure was carried out to minimize the error in estimation of limb orientations.

Kinematic data were collected from the IMUs in order to infer the angle of the hand, relative to the forearm. Oriented with their inherent $x$-axis along the forearm and the $y$-axis aiming medially, IMU $A$ was placed on the hand dorsum and IMU $B$ was located on the forearm (Fig. 7). The $z$-axis of each IMU was orthogonal to the $xy$-plane, forming a right-hand frame.

Gyroscope and accelerometer data were recorded from each IMU at a rate of 28 measurements per second. For each IMU, these data consisted of: time series of the Tait-Bryan angles assembled in a rotation matrix ${}^{I}{R}_{G}$ defined with respect to a global frame $G$, where the superscript *I* denotes the IMU deviec (*A* or *B*); the components of the angular velocity (roll, pitch, and yaw rates); and the $x$-, $y$-, and $z$-components of the gravity vector.

The precise orientation of each IMU with respect to the hand or forearm was calculated through a calibration procedure adapted from^52^. Specifically, the orientation of each IMU was expressed using a constant rotation matrix assembled from the three unit vectors

1 $${}^{I}R_{S}=[{}^{I}x_{S}\quad {}^{I}y_{S}\quad {}^{I}z_{S}],$$

where the subscript $S$ refers to the limb segment where it was placed (hand, $H$, or forearm, $F$). The matrix thus described the orientation of the limb segment with respect to the IMU.

The components of the three unit vectors in (1) were inferred from the calibration phase at the beginning of each experiment, where participants performed a sequence of pronations and supinations while holding the forearm as horizontal as possible with respect to the ground. Within a first approximation, keeping the forearm parallel to the ground leads to pronation and supination developing primarily about the $x$-axis. Given also that roll of the hand relative to the wrist play is not kinematically feasible^53^, the $x$-axes of the IMUs were physiologically aligned. Therefore, the $x$-axis unit vector was taken from the three measurements of the gyroscope as

2 $${}^{I}{x}_{S}=\frac{\omega }{| \omega | },$$

where $\omega$ is the angular velocity vector measured by the IMU. Given undesired motions and experimental noise, the right-hand-side of equation (2) was averaged in time during pronation and supination and then normalized to the unit vector.

Although subjects were instructed in the calibration stage to hold their forearm and hand parallel to the ground as much as possible, it cannot be assumed that the the $z$-axes were exactly normal to the ground. Hence, the $z$-axis was determined from the direction of gravity, $g$, captured by the accelerometer. Thus, the $z$-axis was inferred as

3 $${}^{I}{z}_{S}=\frac{g}{| g| }.$$

Similar to the identification of the $x$-axis, we averaged the measurements from the accelerometer to account for undesired motion and experimental noise during calibration.

The $y$-axis was computed as the cross-product of the other two vectors to form a right-hand orthogonal coordinate system as

4 $${}^{I}y_{S}=\frac{\,{}^{I}z_{S}\times \,{}^{I}x_{S}}{| \,{}^{I}z_{S}\times \,{}^{I}x_{S}| }.$$

Finally, to correct for participants’ inability to hold their forearm precisely horizontal to the ground during calibration, the $z$-axis was recomputed through a cross-product of the expressions of $x$-axis and $y$-axis from equations (2) and (3), and normalized to the unit vector, producing

5 $${}^{I}R_{S}=\left[\,{}^{I}x_{S}\quad \,{}^{I}z_{s}\times \,{}^{I}x_{S}\quad \,{}^{I}x_{S}\times (\,{}^{I}z_{s}\times \,{}^{I}x_{S})\right].$$

The orientations of each IMU relative to its respective limb segment, ${}^{A}{R}_{H}$ and ${}^{B}{R}_{F}$, was assumed to be fixed. Taking the device’s instantaneous orientation with respect to the global frames, ${}^{A}{R}_{G}$ and ${}^{B}{R}_{G}$, the orientation of the hand relative to the forearm was calculated from manipulations of matrices at all times as

6 $${}^{H}R_{F}={({}^{A}R_{H})}^{-1}(\,{}^{A}R_{G})\,{({}^{B}R_{G})}^{-1}({}^{B}R_{F}),$$

where the inverse is equivalent to transposition of the orthogonal rotation matrix. From the time series of the orientations, the time evolution of the wrist flexion-attachment angle were extracted.

**Figure 8:**
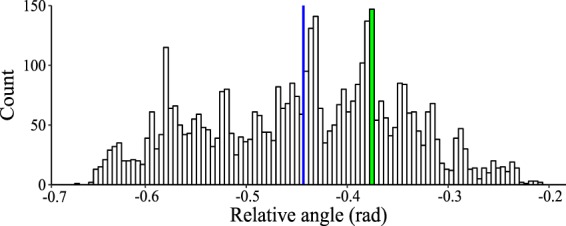
A representative histogram of instantaneous wrist angles recorded during a $157$ seconds-long navigation trial. $4525$ measurements of instantaneous wrist angle were captured, varying from $-0.66$ rad to $-0.20$ rad. This range was uniformly divided into 100 intervals with a range of 0.004 rad and each observation was binned into the interval it belonged to. A count of observations within each interval yielded a probability for each interval. For example, $149$ observations were between $-0.371$ and $-0.367$ rad, highlighted in the green bar. Therefore, the probability of an angle falling under this interval is $3.2$%. This probability contributed to the entropy, measured with Eq. (7). The mean angle ($-0.44$ rad) is represented by the blue vertical line.

Two scores of motor performance were computed for the wrist angle: range of motion and angle variability. Range of motion was taken as the 5th percentile value among instantaneous angles, subtracted from the 95th percentile value^54^. Angle variability was quantified using Shannon entropy^55^, an information-theoretic measure of uncertainty, which for a discrete random variable $X$ takes the following form:

7 $$H(X)=-\sum p(x){\log }_{2}p(x),$$

where $p(x)$ is the probability that the random variable $X$ takes value $x$. A logarithm with base 2 is chosen such that $H(X)$ is given in bits. In our analysis, $X$ is the wrist angle, which was binned into 100 intervals (Fig. 8), and the probability of each bin was computed to estimate the probability mass function.

#### Microsoft kinect data processing

Motor compensation was assessed from the data collected by the Kinect. The Kinect recorded the position of bright blue pixels corresponding to the blue tape. Participants’ movement was recorded at a frame resolution of $640\times 480$ pixels, at a sampling rate of 30 frames per second. For each frame, a line of best fit was approximated using the least squares method. The line equation was translated to an instantaneous trunk angle, defined as the angle between the fitted line and the line normal to the ground. Given a time series of instantaneous trunk angles, the trunk’s range of motion and angle variability were computed similarly to wrist angles.

#### Statistical analysis

Each of the kinematic variables was fitted into a generalized linear mixed-effects model, specifying a Gaussian error family with an identity link^56^. Condition (control or experimental) was specified as an independent variable, and navigation trial and user identity were specified as random effects. Each model was tested for significance by comparing it against a null model using a likelihood ratio test.

Survey questions were administered to gauge the influence of retrofitting on preference between the two conditions, their level of enjoyment, and likelihood to repeat the task. All 7-scale Likert scores were transformed and standardized to values between 0 and 1. For the first two questions, a one-sample t-test was performed, comparing each mean score against 0.5. For the latter two questions, a two-sample t-test was performed, comparing the mean scores against one another.

For all statistical tests, the level of significance was set to $\alpha =0.05$.

## Results

Volunteers’ wrist and trunk movements were compared between the experimental condition where they executed the motor task with the 3D-printed retrofit attachment, and the control condition where performed the same without the retrofit attachment.

**Figure 9:**
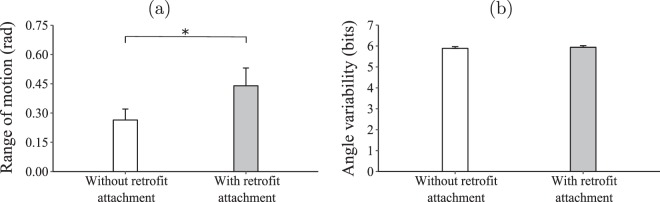
The impact of retrofitting on wrist range of motion (**a**) and angle variability. (**b**) The bars represent means and the error bars represent standard error. The asterisk indicates statistically different means.

In four trials, one of the IMUs disconnected in the middle of a trial due to abrupt and forceful movement of the user. Therefore, only 21 of IMU data-sets contained a complete recording of both conditions and were retained for statistical analysis. For these users, a significantly larger range of motion was observed when users manipulated the Novint Falcon with the retrofit attachment, relative to when they operated it without the attachment (${\chi }_{1}^{2}=7.420,\,p=0.006$; Fig. 9a). The users spanned the range of motion similarly, reflected by indistinguishable angle variability (${\chi }_{1}^{2}=0.286,\,p=0.592$; Fig. 9b).

**Figure 10:**
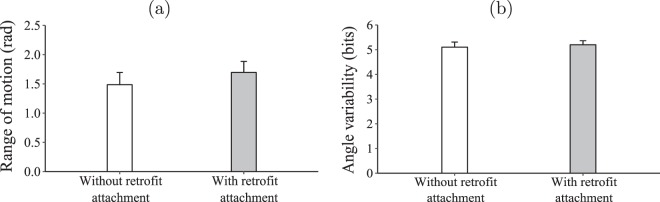
The impact of retrofitting on trunk range of motion (**a**) and angle variability. (**b**) The bars represent means and the error bars represent standard error.

For trunk movement, all data-sets were retained for statistical analysis. We failed to find a significant difference in the range of motion of participants’ trunks among conditions (${\chi }_{1}^{2}=1.754,\,p=0.185$; Fig. 10a). Similarly, a significant difference was not registered when examining their trunks’ angle variability, either (${\chi }_{1}^{2}=0.443,\,p=0.505$; Fig. 10b).

All users’ survey responses were analyzed. Participants indicated that they enjoyed the experimental condition more than the control condition ($t=2.309,\,df=24,\,p=0.029$), and that they would preferred using the Novint Falcon with the retrofit attachment rather than without it ($t=2.886,\,df=24,\,p=0.008$). Participants were also more likely to repeat the exercise with the enhanced Novint Falcon rather than the unmodified device ($t=3.536,\,df=47,\,p < 0.001$).

## Discussion

Neuromuscular disability often disrupts patients’ participation in activities of daily living, preventing them from being productive members of society^2^. Recovery of neuromuscular function is well-documented, but it requires adherence to a rehabilitation regimen consisting of repetitive, high-intensity exercises^4–6^. However, logistical barriers, lack of medical specialists, and economic strains deny the majority of patients from receiving adequate treatment, thereby hindering their recovery.

Low-cost gaming systems are increasingly being repurposed for applications in telerehabilitation, to deliver physical therapy to patients’ homes and drive down the costs of treatment. Their affordability and portability can particularly benefit disadvantaged patient populations who cannot access quality medical care due to cost or geography^7–9^. By performing rehabilitative exercise at their convenience, patients can regain motor skills faster and accelerate their recovery toward reintegration into society. However, the non-universal design of game controllers diminishes the extent to which patients can enjoy from their rehabilitative features. From a physical therapy perspective, patients may struggle with adapting their hand posture to grasp a commercial end-effector, whose design is aimed at healthy users. Moreover, game controllers can train only a subset of possible joint movements and therefore treat a narrow range of pathologies. From a motivational point of view, a game controller facilitates a few movements, which would be monotonous and uninteresting to use in the long run.

Additive manufacturing offers a unique opportunity to enable alternative interfacing with these game controllers. We propose to leverage the versatility of 3D printing to create retrofit attachments for commercial game controllers. The role of these attachments is threefold. First, retrofit attachments could enable different postures toward a targeted intervention, based on patients’ pathology. Second, with a set of retrofit attachments, patients may receive a multi-faceted treatment, aiming at multiple impairments. Third, retrofit attachments could diversify the rehabilitation regimen and increase the motivation to perform exercise. In this study, we explored the feasibility of this approach with a custom attachment for a haptic device. Our results suggest that retrofit attachments can fulfill all three roles and mitigate the physical and motivational limitations of game controllers

We observed that the retrofit attachment successfully broadened participants’ range of motion. The attachment did not influence the variability of the wrist angle, suggesting that it scaled the movement but did not increase or decrease the extent to which the wrist spans its range of motion. Based on these findings, patients undergoing fine motor rehabilitation could benefit from using the retrofit attachment. For example, many stroke survivors are left with disabling spasticity and are prescribed with fine motor exercises for the fingers and wrist^57–59^. Since true motor recovery is associated with kinematics similar to those of healthy individuals, increasing these patients’ wrist range of motion is essential for reinforcing motor learning and restoring elemental motor behaviors of the hand^60–62^.

The findings of this study have important implications for motivation as well. Patients often fail to adhere to their regimen due to the unengaging nature of the exercise performed during rehabilitation^17^. Diversification of their exercise regimen by means of 3D-printed attachments could break the boring and repetitive rehabilitation paradigm. Supporting this notion, participants expressed preference toward the retrofit attachment and indicated that they enjoyed the activity more when they were using the enhanced end-effector. Furthermore, participants indicated that they are more likely to repeat the exercise if performed with a retrofit controller. In this manner, retrofitting game controllers could boost the motivation to perform exercise, augment adherence, and ultimately, improve rehabilitation outcomes.

In addition to wrist movement, the impact of the retrofit attachment on trunk movement was investigated to ensure that its use did not elicit compensatory movements^17^. When implementing a compensatory strategy, patients recruit body parts that are not normally involved in the movement to add degrees of freedom. For example, stroke patients tend to displace their trunk during reaching tasks to compensate for limited movement of their arm^26,27,61^. These movements are energetically inefficient and hinder the functional recovery of the affected limb^18^. We tested whether the use of the attachment caused users to compensate for their inability to execute a motion by moving their trunk. The attachment did not increase nor reduce compensatory movement. Therefore, it is capable of enhancing wrist movement without exacerbating or minimizing non-physiological movements.

In the case one is still interested in addressing compensatory movements, alternative attachment designs can be explored. Custom attachments could isolate joint movements by restricting certain joints. For example, an attachment that constrains forearm movement relative to the upper arm could promote isolated joint movement of the wrist. More elaborate attachments could constrain shoulder attachment or trunk bending. Even higher levels of control and personalization may be achieved by scanning patients and fitting attachments to their body parts and posture^63,64^.

The use of additive manufacturing is principle to our proposed approach. With the flexibility of 3D printing technologies, one can create structures with versatile geometries and different materials. The spectrum of movements game controllers support can be expanded to diversify patients’ rehabilitation regimens. Conceivably, a single gaming system could be modified with a set of retrofit attachments to relay task-related exercises^65^ and bimanual training^66^. Such a multi-faceted home therapy will help patients recover a broader range of functional movements and attain their full rehabilitation potential. Furthermore, the increasing ubiquity and affordability of 3D printers facilitates this vision. Retrofit attachments can be produced virtually from anywhere^67,68^: private businesses, clinics, and patients’ homes, thereby increasing the accessibility of quality home therapy.

Although our findings support the viability of the retrofitting approach, a number of limitations are acknowledged. Primarily, the participants in this study were not patients. This limitation was addressed in part by the implementation of standard techniques from disability awareness training programs^42,48,49^. However, the simulated disability does not replicate the one caused by a true profound impairment. To ensure that retrofit attachments offer benefits with no adverse outcomes, their usability must be explored in a controlled setting with patients undergoing rehabilitation. These clinical studies could also investigate the motivational impact of retrofit attachments. While survey responses in this study suggest that users would be more motivated to perform exercise with a retrofit controller, participants are not expected to adhere to a rehabilitation regimen over time and performing the exercise does not have direct implication on their function and health. Therefore, their intent of adoption may not reflect that of patients. A longitudinal study with patients could elucidate the influence of 3D printing retrofit on motivation, adoption, and adherence, along with its safety. Finally, since the survey questions were subjective in nature and the participants had been briefed by the experimenter prior to answering them, we acknowledge the possibility of a bias. In this vein, administering a blind survey, with debriefing instead of a briefing, would have strengthened our findings with respect to motivation.

We demonstrated the possibility of supplementing a haptic device with a 3D-printed retrofit attachment to enhance patients’ motor performance and motivation in game controller-mediated rehabilitation. Our results indicate that the integration of the attachment into rehabilitative exercise has the potential to encourage wrist joint movements while maintaining the same level of motor compensation. The introduction of retrofit attachments could also increase participants’ satisfaction and intention to repeat the exercise, thereby improving adherence to the rehabilitation regimen. Nonetheless, a longitudinal study with patients is warranted to validate these propositions in a medical setting. If proven clinically useful, the retrofit approach could be generalized to almost any game controller to target a variety of joints. The implementation of the approach is expected to accelerate the recovery of patients’ motor skills, and ultimately, improve their quality of life.

### Ethics approval and informed consent

All methods were performed in accordance with the relevant guidelines and regulations set by New York University’s institutional review board, the University Committee on Activities Involving Human Subjects (UCAIHS; IRB#FY2019-2828). Informed consent for participation was obtained from all subjects.

## Supplementary information

Supplementary Information.

## Data Availability

Datasets and codes used in the analyses are stored at the authors’ home institution and will be provided upon request, in compliance with New York University’s institutional review board.
